# Supplementary material for: Mediolateral foot placement control can be trained: Older adults learn to walk more stable, when ankle moments are constrained
Source: PLoS One. 2023 Nov 1;18(11):e0292449. doi: 10.1371/journal.pone.0292449 (PMC10619794; doi:10.1371/journal.pone.0292449)
Supplement: S1 Fig — (PDF) [file pone.0292449.s001.pdf]

S1 SPPB Four meter walk test

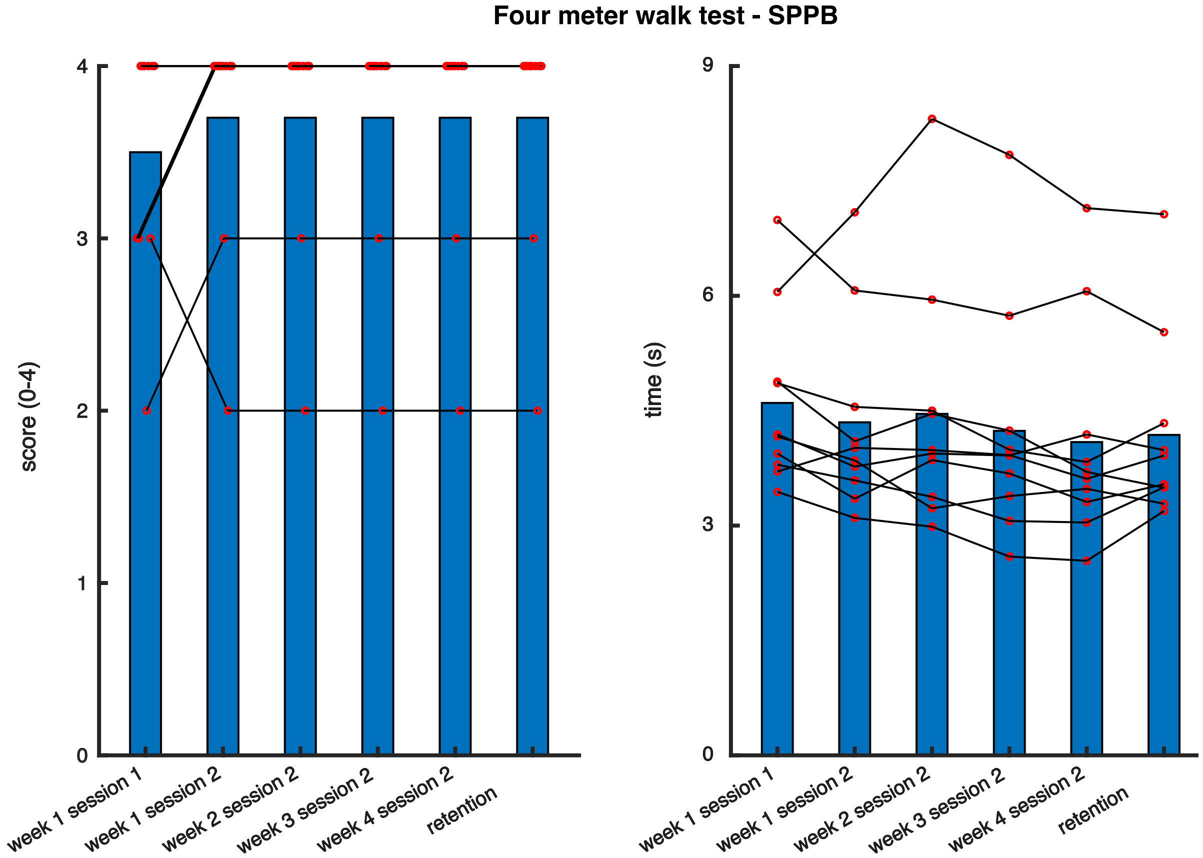

S1 Fig 1. Four-meter walking test of the Short Physical Performance Battery (SPPB). SPPB scores (left panel) and time in seconds (right panel) have been depicted. Red circles represent individual data points.
