# Supplementary material for: Mediolateral foot placement control can be trained: Older adults learn to walk more stable, when ankle moments are constrained
Source: PLoS One. 2023 Nov 1;18(11):e0292449. doi: 10.1371/journal.pone.0292449 (PMC10619794; doi:10.1371/journal.pone.0292449)
Supplement: S1 File — (PDF) [file pone.0292449.s002.pdf]

S2 Tables main analysis statistical tests

In supplementary material 2, we report the tables from the statistical tests computed in JASP, for our clinical and biomechanical outcome measures. **Significant effects** have been printed in **bold**.

Clinical outcome measures – Training effect

SPPB

Paired Samples T-Test

Measure 1		Measure 2	t	df	p
baseline	-	last training session	-1.078	9	0.309

Note. Student's t-test.

Descriptives

Descriptives

	N	Mean	SD	SE
baseline	10	11.200	1.135	0.359
last training session	10	11.600	0.843	0.267

FES-I

Paired Samples T-Test

Measure 1		Measure 2	t	df	p
baseline	-	last training session	-0.452	9	0.662

Note. Student's t-test.

Descriptives

Descriptives

	N	Mean	SD	SE
baseline	10	18.550	2.455	0.776
last training session	10	18.900	2.331	0.737

Biomechanical outcome measures – Training effect

Degree of foot placement control - Normal walking

Repeated Measures ANOVA

Within Subjects Effects

Cases	Sum of Squares	df	Mean Square	F	p
Week	0.001	3	4.620e-4	0.099	0.960
Residuals	0.126	27	0.005		
Session	0.006	1	0.006	0.423	0.532
Residuals	0.127	9	0.014		
Week ✻ Session	0.067	3	0.022	5.112	**0.006**
Residuals	0.118	27	0.004		

Note. Type III Sum of Squares

Post Hoc Tests

Post Hoc Comparisons - Week ✻ Session

		Mean Difference	SE	t	Cohen's d	p_{bonf}
1, 1	2, 1	0.005	0.030	0.182	0.016	1.000
	3, 1	0.060	0.030	1.991	0.171	1.000
	4, 1	-0.023	0.030	-0.756	-0.065	1.000
	1, 2	0.028	0.037	0.762	0.080	1.000
	2, 2	0.027	0.037	0.736	0.078	1.000
	3, 2	-0.012	0.037	-0.312	-0.033	1.000
	4, 2	0.068	0.037	1.818	0.194	1.000
2, 1	3, 1	0.054	0.030	1.810	0.155	1.000
	4, 1	-0.028	0.030	-0.937	-0.080	1.000
	1, 2	0.023	0.037	0.607	0.065	1.000
	2, 2	0.022	0.037	0.596	0.063	1.000
	3, 2	-0.017	0.037	-0.458	-0.049	1.000
	4, 2	0.062	0.037	1.672	0.178	1.000
3, 1	4, 1	-0.082	0.030	-2.747	-0.236	0.228
	1, 2	-0.032	0.037	-0.852	-0.091	1.000
	2, 2	-0.032	0.037	-0.869	-0.092	1.000
	3, 2	-0.071	0.037	-1.938	-0.204	1.000
	4, 2	0.008	0.037	0.214	0.023	1.000
4, 1	1, 2	0.051	0.037	1.362	0.145	1.000
	2, 2	0.050	0.037	1.345	0.143	1.000
	3, 2	0.011	0.037	0.297	0.032	1.000
	4, 2	0.090	0.037	2.455	0.258	0.591
1, 2	2, 2	-6.343e-4	0.030	-0.021	-0.002	1.000
	3, 2	-0.040	0.030	-1.321	-0.113	1.000
	4, 2	0.040	0.030	1.322	0.113	1.000
2, 2	3, 2	-0.039	0.030	-1.300	-0.112	1.000
	4, 2	0.040	0.030	1.343	0.115	1.000

Post Hoc Comparisons - Week ✻ Session

	Mean Difference	SE	t	Cohen's d	p_{bonf}
3, 2 4, 2	0.079	0.030	2.643	0.227	0.300

Note. P-value adjusted for comparing a family of 28

Degree of foot placement control – Training condition

Repeated Measures ANOVA
Within Subjects Effects

Cases	Sum of Squares	df	Mean Square	F	p
Week	0.011	2	0.005	0.278	0.760
Residuals	0.350	18	0.019		
Session	0.019	1	0.019	3.221	0.106
Residuals	0.054	9	0.006		
Week ✻ Session	0.015	2	0.008	1.489	0.252
Residuals	0.093	18	0.005		

Magnitude of foot placement error - Normal walking

Repeated Measures ANOVA
Within Subjects Effects

Cases	Sum of Squares	df	Mean Square	F	p
Week	3.226e-5 [a]	3 [a]	1.075e-5 [a]	6.969 [a]	**0.001** [a]
Residuals	4.166e-5	27	1.543e-6		
Session	1.198e-6	1	1.198e-6	3.828	0.082
Residuals	2.817e-6	9	3.130e-7		
Week ✻ Session	5.080e-7	3	1.693e-7	0.706	0.557
Residuals	6.479e-6	27	2.400e-7		

Note. Type III Sum of Squares

[a] Mauchly's test of sphericity indicates that the assumption of sphericity is violated ($p < .05$).

Post Hoc Tests

Post Hoc Comparisons - Week

		Mean Difference	SE	t	Cohen's d	p_{bonf}
1	2	0.001	3.928e-4	3.222	0.686	**0.020**
	3	0.001	3.928e-4	3.661	0.780	**0.006**
	4	0.002	3.928e-4	4.106	0.875	**0.002**
2	3	1.723e-4	3.928e-4	0.439	0.093	1.000
	4	3.470e-4	3.928e-4	0.883	0.188	1.000
3	4	1.747e-4	3.928e-4	0.445	0.095	1.000

Note. P-value adjusted for comparing a family of 6

Note. Results are averaged over the levels of: Session

Magnitude of foot placement error – Training condition

Repeated Measures ANOVA
Within Subjects Effects

Cases	Sum of Squares	df	Mean Square	F	p
Week	2.144e-6	2	1.072e-6	1.120	0.348
Residuals	1.723e-5	18	9.570e-7		
Session	3.827e-6	1	3.827e-6	5.545	**0.043**
Residuals	6.211e-6	9	6.901e-7		
Week ✻ Session	1.458e-6	2	7.288e-7	2.926	0.079
Residuals	4.484e-6	18	2.491e-7		

Note. Type III Sum of Squares

Post Hoc Tests
Post Hoc Comparisons - Session

		Mean Difference	SE	t	Cohen's d	p_{bonf}
1	2	5.051e-4	2.145e-4	2.355	0.394	**0.043**

Note. Results are averaged over the levels of: Week

Gait stability - Normal walking

Repeated Measures ANOVA
Within Subjects Effects

Cases	Sum of Squares	df	Mean Square	F	p
Week	0.018	3	0.006	6.624	**0.002**
Residuals	0.024	27	8.835e-4		
Session	0.002	1	0.002	6.293	**0.033**
Residuals	0.003	9	3.120e-4		
Week ✻ Session	1.399e-4	3	4.664e-5	0.087	0.967
Residuals	0.015	27	5.378e-4		

Note. Type III Sum of Squares

Post Hoc Tests
Post Hoc Comparisons - Week

		Mean Difference	SE	t	Cohen's d	p_{bonf}
1	2	0.021	0.009	2.216	0.250	0.212
	3	0.039	0.009	4.123	0.466	**0.002**
	4	0.033	0.009	3.490	0.394	**0.010**
2	3	0.018	0.009	1.906	0.215	0.404
	4	0.012	0.009	1.273	0.144	1.000
3	4	-0.006	0.009	-0.633	-0.072	1.000

Note. P-value adjusted for comparing a family of 6
Note. Results are averaged over the levels of: Session

Post Hoc Comparisons - Session

	Mean Difference	SE	t	Cohen's d	p_{bonf}
1 2	0.010	0.004	2.509	0.119	**0.033**

Note. Results are averaged over the levels of: Week

Gait stability – Training condition

Repeated Measures ANOVA
Within Subjects Effects

Cases	Sum of Squares	df	Mean Square	F	p
Week	0.005	2	0.002	1.052	0.370
Residuals	0.039	18	0.002		
Session	1.965e-4	1	1.965e-4	0.337	0.576
Residuals	0.005	9	5.838e-4		
Week ✻ Session	9.777e-6	2	4.888e-6	0.014	0.986
Residuals	0.006	18	3.543e-4		

Note. Type III Sum of Squares

Step width - Normal walking

Repeated Measures ANOVA
Within Subjects Effects

Cases	Sum of Squares	df	Mean Square	F	p
Week	0.002	3	6.089e-4	5.319	**0.005**
Residuals	0.003	27	1.145e-4		
Session	8.588e-5	1	8.588e-5	0.615	0.453
Residuals	0.001	9	1.396e-4		
Week ✻ Session	1.615e-4	3	5.385e-5	0.517	0.674
Residuals	0.003	27	1.041e-4		

Note. Type III Sum of Squares

Post Hoc Tests
Post Hoc Comparisons - Week

		Mean Difference	SE	t	Cohen's d	p_{bonf}
1	2	0.013	0.003	3.893	0.410	**0.004**
	3	0.004	0.003	1.189	0.125	1.000
	4	0.006	0.003	1.847	0.195	0.455
2	3	-0.009	0.003	-2.704	-0.285	0.070
	4	-0.007	0.003	-2.046	-0.216	0.304
3	4	0.002	0.003	0.658	0.069	1.000

Note. P-value adjusted for comparing a family of 6
Note. Results are averaged over the levels of: Session

Step width – Training condition

Repeated Measures ANOVA

Within Subjects Effects

Cases	Sum of Squares	df	Mean Square	F	p
Week	8.954e-4 [a]	2 [a]	4.477e-4 [a]	1.355 [a]	0.283 [a]
Residuals	0.006	18	3.305e-4		
Session	0.002	1	0.002	3.936	0.079
Residuals	0.004	9	4.138e-4		
Week ✻ Session	1.222e-4	2	6.109e-5	0.540	0.592
Residuals	0.002	18	1.132e-4		

Note. Type III Sum of Squares

[a] Mauchly's test of sphericity indicates that the assumption of sphericity is violated ($p < .05$).

Stride time - Normal walking

Repeated Measures ANOVA
Within Subjects Effects

Cases	Sum of Squares	df	Mean Square	F	p
Week	0.010 [a]	3 [a]	0.003 [a]	0.589 [a]	0.627 [a]
Residuals	0.145	27	0.005		
Session	0.002	1	0.002	1.087	0.324
Residuals	0.018	9	0.002		
Week ✻ Session	5.297e-4 [a]	3 [a]	1.766e-4 [a]	0.123 [a]	0.946 [a]
Residuals	0.039	27	0.001		

Note. Type III Sum of Squares

[a] Mauchly's test of sphericity indicates that the assumption of sphericity is violated ($p < .05$).

Stride time – Training condition

Repeated Measures ANOVA
Within Subjects Effects

Cases	Sum of Squares	df	Mean Square	F	p
Week	0.008	2	0.004	2.809	0.087
Residuals	0.027	18	0.001		
Session	0.003	1	0.003	2.966	0.119
Residuals	0.009	9	0.001		
Week ✻ Session	3.846e-4	2	1.923e-4	0.410	0.669
Residuals	0.008	18	4.685e-4		

Note. Type III Sum of Squares

Retention

Magnitude of foot placement error

Paired Samples T-Test

Measure 1		Measure 2	t	df	p	Cohen's d
w1_s1_baseline	-	retention_normal	2.700	9	**0.024**	0.854
w4_s2_training	-	retention_LesSchuh	2.124	9	0.063	0.672

Note. Student's t-test.

Gait stability

Paired Samples T-Test

Measure 1		Measure 2	t	df	p	Cohen's d
w1_s1_baseline	-	retention_normal	1.821	9	0.102	0.576

Note. Student's t-test.
