# Supplementary material for: Mediolateral foot placement control can be trained: Older adults learn to walk more stable, when ankle moments are constrained
Source: PLoS One. 2023 Nov 1;18(11):e0292449. doi: 10.1371/journal.pone.0292449 (PMC10619794; doi:10.1371/journal.pone.0292449)
Supplement: S2 File — (PDF) [file pone.0292449.s003.pdf]

S3 Within session analyses

In S3, we assess the outcome measures at similar data points as in Hoogstad, van Leeuwen (21), (i.e. based on blocks of 30 strides). In this way, we aim to give more insight in how any improvements over time manifested themselves. First, we looked for immediate effects of LesSchuh (comparing normal walking end to training start). Second, we evaluated changes throughout the training (comparing training start to training end). Third, we considered after-effects (comparing normal walking end to after-effect start). Lastly, we assessed whether any after-effect washed out over time (comparing after-effect start to after-effect end).

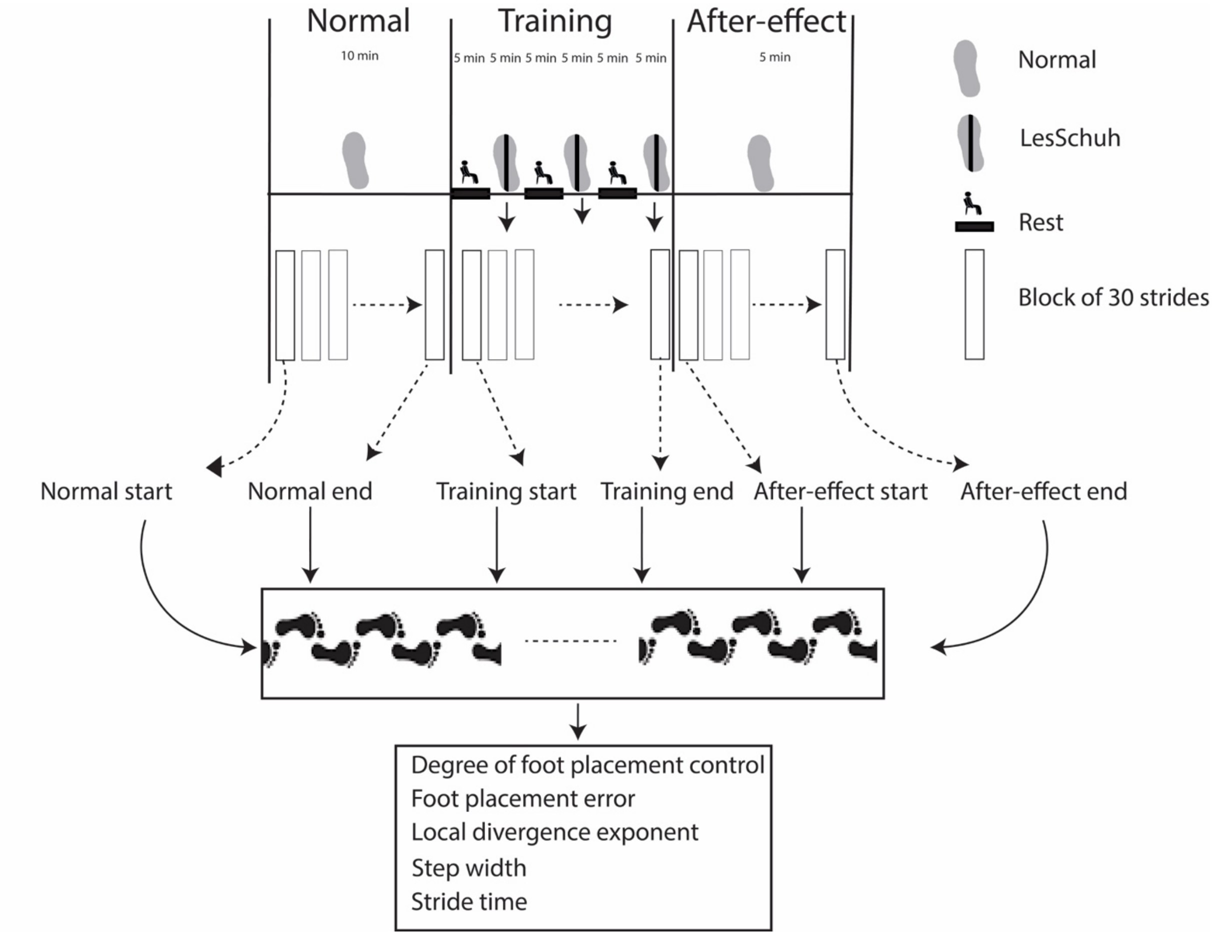

S3 Fig 1. Flow of data processing.

Immediate effect of walking with ankle moment constraints

For evaluating the immediate effect of walking with constrained ankle moments, we performed a repeated-measures ANOVA with the factors Condition ("normal walking end" vs "training start"), Week ("1","2","3","4") and Session ("1", "2"). If the factor Condition, or its interactions, were significant, the repeated-measures ANOVA was followed up by Bonferroni corrected post-hoc t-tests to compare normal walking end to training start.

Compliance with instructions

We computed mean toeing-out angles, to evaluate whether participants complied with the instruction to keep their feet pointing straight ahead, avoiding a toeing-out strategy (25).

Participants were instructed to point their feet straight ahead while walking with LesSchuh. Yet, when testing for immediate effects on toe-out angles, we found a significant Condition*Session interaction. We averaged across week and performed two Bonferonni corrected t-tests to explore this interaction effect. No significant differences were found, indicating that on average the participants complied with our instructions during the first part of the training.

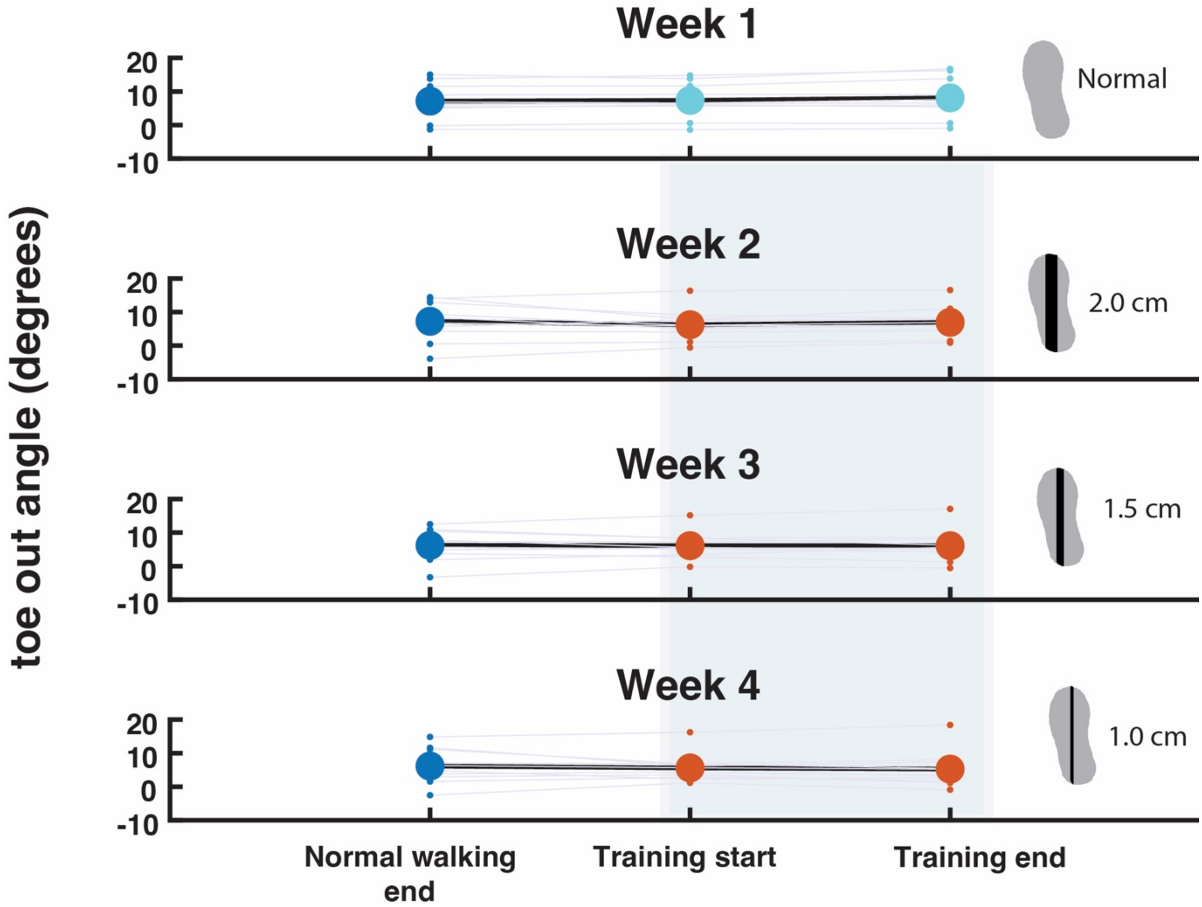

S3 Fig 2. Toe-out angles. *The angles are averaged across sessions 1 and 2. Positive angles represent more toeing out, whereas negative angles represent more toeing in. Participants did not significantly alter their toe-out angle when walking with LesSchuh (Shaded blue area with red dots) as compared to when walking with normal shoes (blue dots).*

Degree of foot placement control

For the relative explained variance (R^2), we found no significant effects for Condition, Week or Session, nor for their interactions (*p>0.05)*.

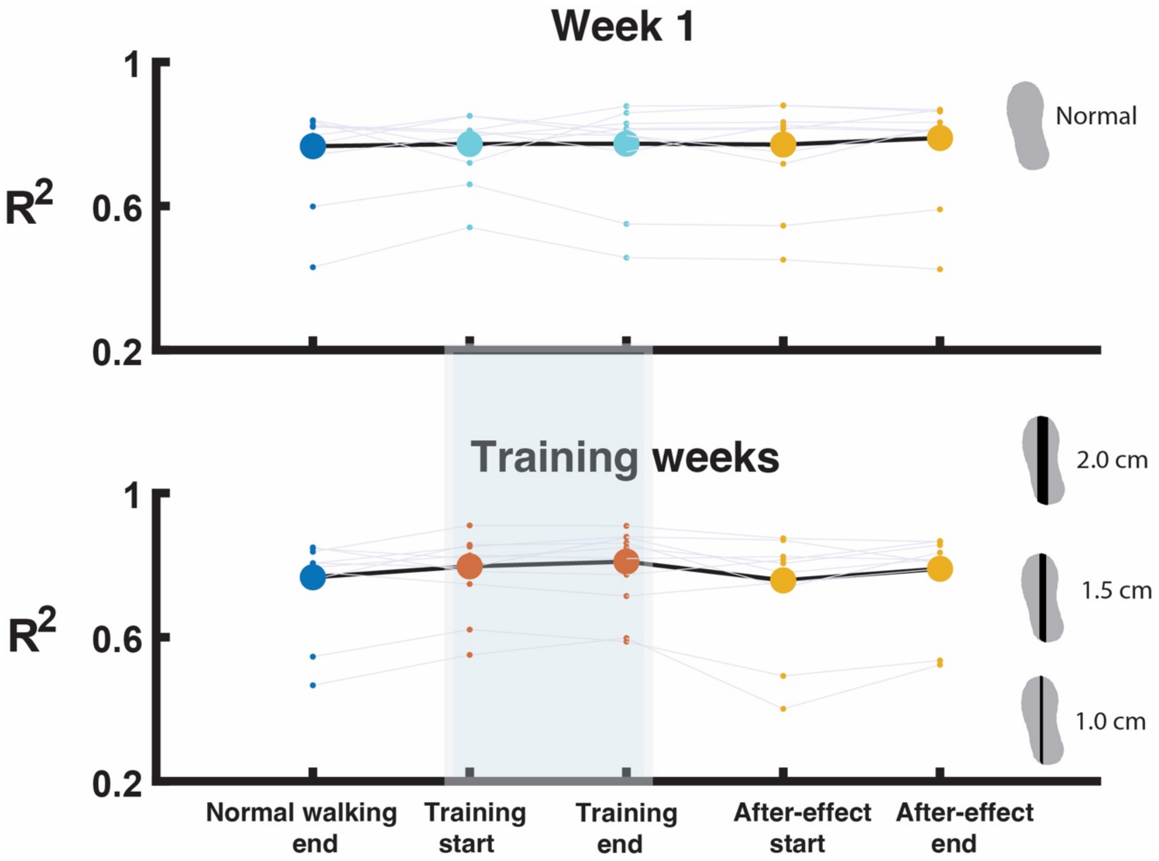

S3 Fig 3. Foot placement control quantified as the relative explained variance of the foot placement model (Model 1). The R^2s were averaged across sessions 1 and 2. In the lower panel the R^2s were also averaged across all training weeks (weeks 2,3 and 4). The shaded blue area with red dots represents the data when participants walked with LesSchuh.

Magnitude of foot placement error

For the magnitude of foot placement error (i.e. the residual of Model 1), we found a significant effect of Condition and of the interaction Condition*Week (*$p<0.05$*). So, we averaged across sessions, and performed four Bonferroni corrected post-hoc t-tests to investigate the effects. In the control week (week 1) and while walking with a two-centimeter ridge (week 2) foot placement error did not significantly change between normal walking end and training start. However, when walking with 1.5- and 1-centimeter ridges (weeks 3 and 4) the foot placement error significantly increased when walking with constrained ankle moments (*$p<0.0125$*).

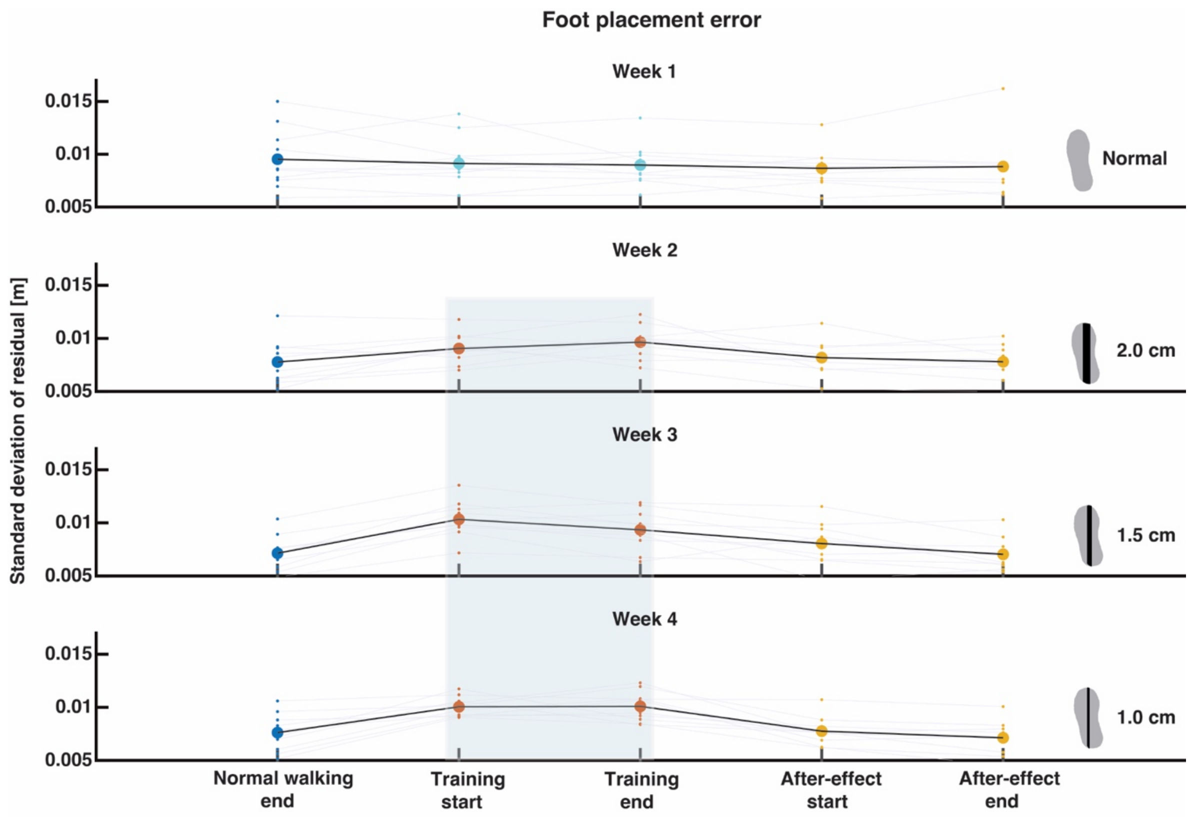

S3 Fig 4. Foot placement error quantified as standard deviation of the foot placement model (Model 1). The foot placements errors were averaged across sessions 1 and 2. The shaded blue area with red dots represents the data when participants walked with LesSchuh.

Gait stability

For the local divergence exponent, we found a significant effect of Condition, Session and the interaction Condition*Week (*$p<0.05$*). Since any immediate effect of LesSchuh concerns the factor Condition, but not Session, we further investigated the Condition*Week interaction by averaging across sessions and computing four Bonferroni corrected post-hoc t-tests. As expected, when comparing the end of the normal walking condition to the start of the training condition, the local divergence exponents significantly increased ($p<0.0125$) during the training weeks (weeks 2-4), as opposed to in the control week (week 1) ($p>0.0125$). This indicated that stability immediately decreased when walking with constrained ankle moments.

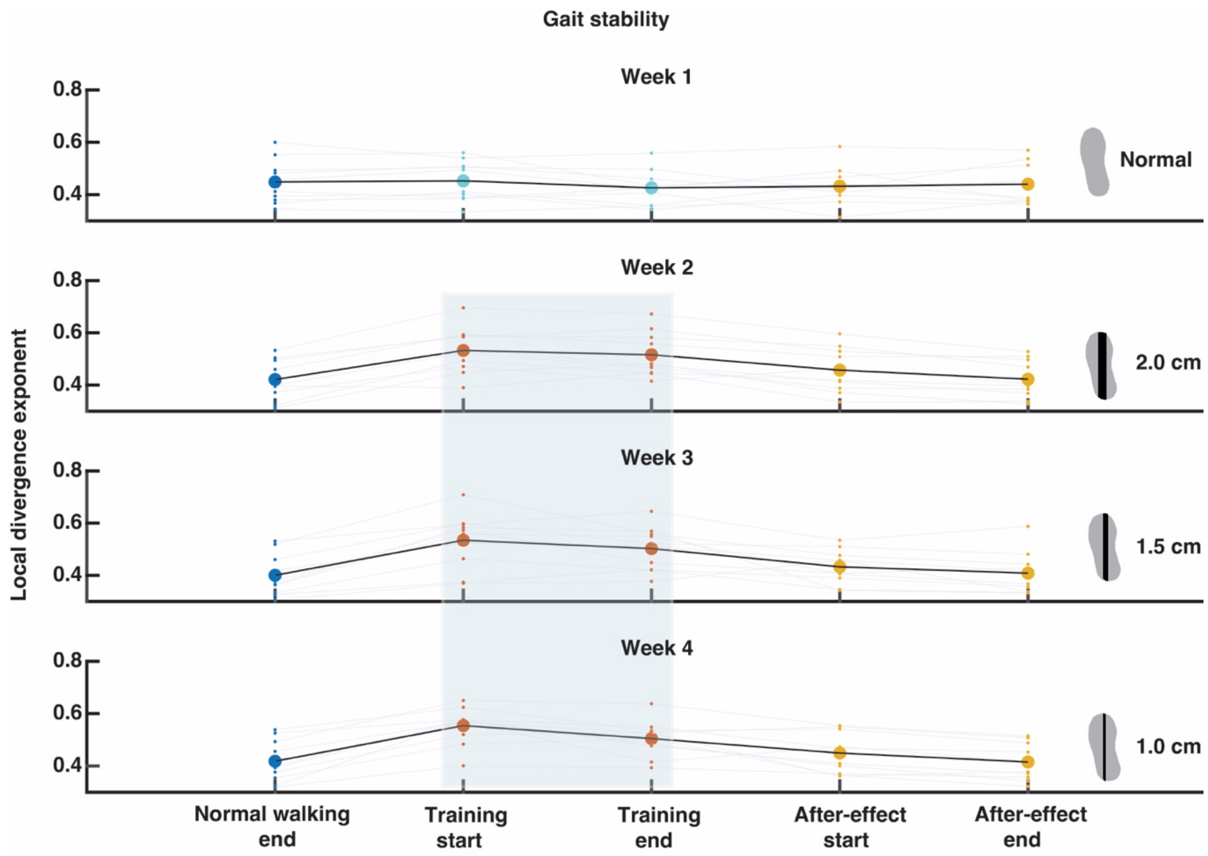

S3 Fig 5. Short term local divergence exponent. The lower the divergence, the more stable the gait pattern. The local divergence exponents were averaged across sessions 1 and 2. The shaded blue area with red dots represents the data when participants walked with LesSchuh.

Step width

For step width Condition, Session and the interaction Condition*Week were significant (*p<0.05*). Since any immediate effect of LesSchuh concerns the effect of Condition, but not the effect of Session, we further investigated the Condition*Week interaction by averaging across sessions and computing four Bonferroni corrected post-hoc t-tests. Although in S3 Fig 6, when comparing training start to normal walking end, step width seemed to increase in the training weeks (weeks 2-4), as opposed to a decrease in the control week (week 1). These changes in step width were not significant (*p>0.0125*).

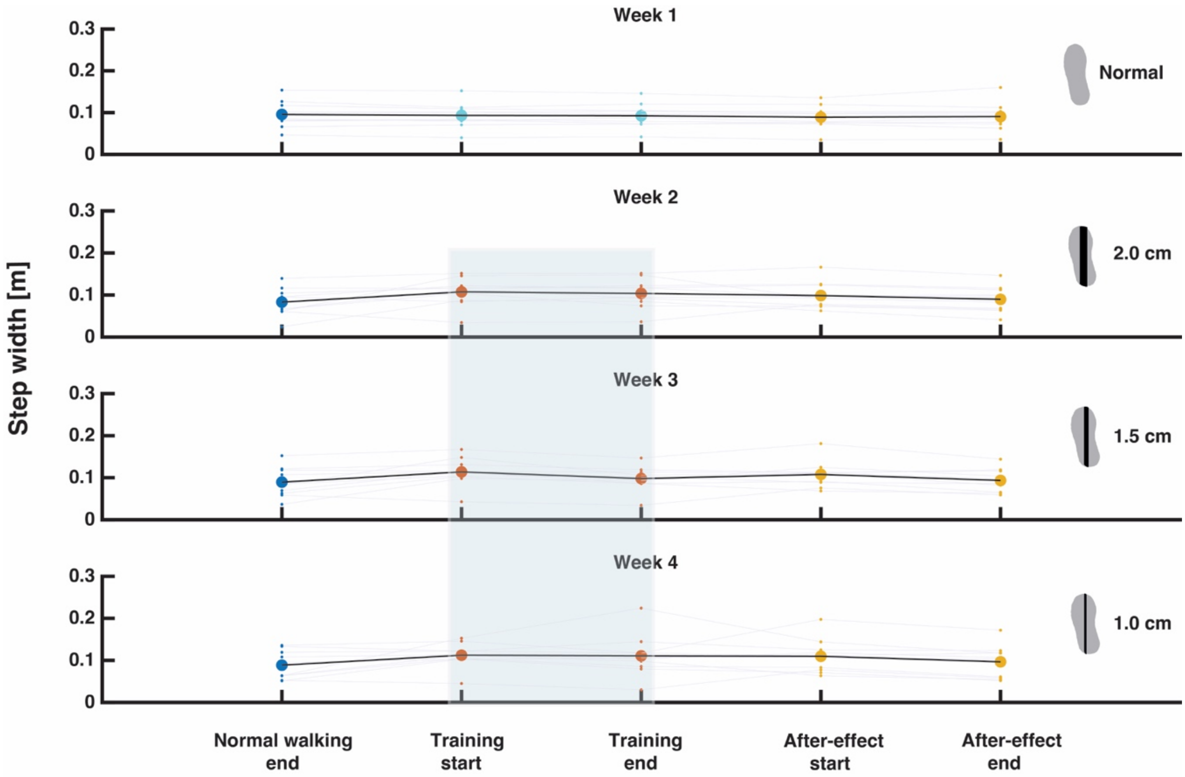

S3 Fig 6. Step width. Step width in meters averaged across sessions 1 and 2. The shaded blue area with red dots represents the data when participants walked with LesSchuh.

Stride time

For stride time we found a significant interaction effect of Condition*Week. So, we averaged across sessions and performed four Bonferroni corrected post-hoc t-tests. Although from S3 Fig 7 it can be observed that, when comparing training start to normal walking end, the control week (week 1) stride time slightly increased, whilst in the training weeks (weeks 2-4) it decreased, these changes in stride time were not significant (*$p>0.0125$*).

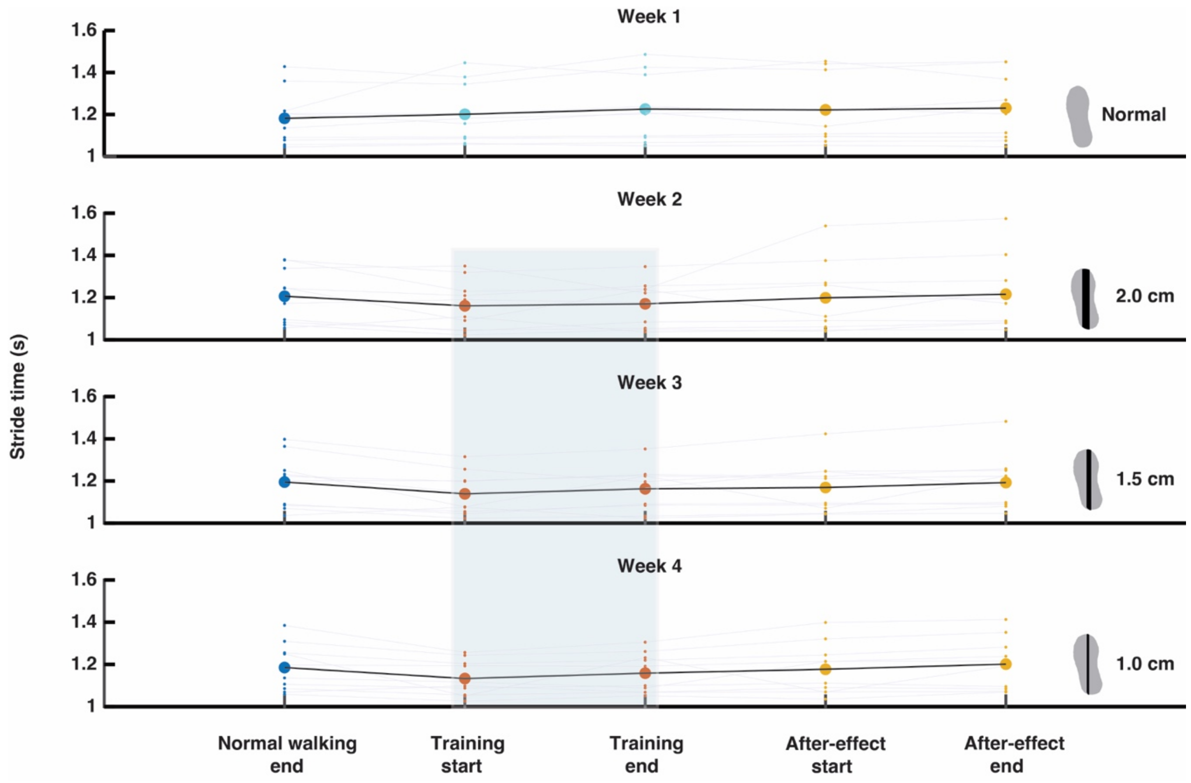

S3 Fig 7. Stride time. Stride time in seconds averaged across sessions 1 and 2. The shaded blue area with red dots represents the data when participants walked with LesSchuh.

Changes throughout the training

To assess the changes in foot placement control throughout the training, we performed a repeated-measures ANOVA with the factors Condition ("training start" vs "training end"), Week ("1","2","3","4") and Session ("1", "2"). If the factor Condition, or its interactions, were significant, the repeated-measures ANOVA was followed up by Bonferroni corrected post-hoc t-tests to test training end against training start.

Compliance with instructions

Throughout the training there was no significant effect of Week, Condition, Session, nor of their interactions on toe-out angle (S3 Fig 2). As there was no effect of Condition, participants did not change their compliance with the instructions throughout the training condition.

Degree of foot placement control

When comparing the end to the start of the training conditions across weeks and sessions, we found a significant interaction of Week*Session (*$p<0.05$*). Since there was no significant effect of Condition nor its interactions, it seems the degree of foot placement control did not improve within each single training condition (S3 Fig 3).

Magnitude of foot placement error

For foot placement error we did not find a significant effect from start to end of a training condition either, nor were there significant effects of Session nor Week (S3 Fig 4).

Gait stability

We found a significant effect of Condition and Week for the local divergence exponents ($p<0.05$). The effect of Condition indicates that throughout the training the local divergence exponent decreased (S3 Fig 5), indicating participants became more stable over time within each training session, not only when walking with LesSchuh, but also in the control week.

Step width

We did not find any significant effects on step width throughout the training condition, nor of Week or Session (S3 Fig 6).

Stride time

We only found a significant effect of Week on stride time. Without a significant effect of Condition, it seems stride time did not change within a single training condition (S3 Fig 7).

After-effects

To test whether there were any after-effects upon returning to walk on normal shoes, we performed a repeated-measures ANOVA with the factors Condition ("normal walking end" vs "after-effect start"), Week ("1","2","3","4") and Session ("1","2"). If the factor Condition, or its interactions, were significant, the repeated-measures ANOVA was followed up by Bonferroni post-hoc t-tests to test between the start of the after-effect condition and normal walking end.

Degree of foot placement control

For the degree of foot placement control, we found a significant interaction effect of Condition*Week*Session. As such, we performed eight Bonferroni corrected post-hoc t-tests, testing for the effect of condition for each week and session. For none of the sessions there was a significant change in foot placement control. Thus, we did not find an after-effect in foot placement control (S3 Fig 3).

Magnitude of foot placement error

When looking at the foot placement error, we found a significant effect of Week, Session and the interaction of Week*Session, but not of Condition. So, we did not find an after-effect in foot placement error (S3 Fig 4).

Gait stability

For stability we found a significant interaction effect between Condition* Week. We averaged across sessions and performed four Bonferoni corrected t-tests. Only in week two we found a significant aftereffect compared to the normal walking condition ($p<0.125$), yet this effect was in the opposite as expected, since stability decreased (S3 Fig 5).

Step width

For step width, we found significant effects of Condition and the interaction Condition*Week. Therefore, we performed four Bonferroni corrected t-tests. Only for the first training week (week 2), step width significantly increased in the aftereffect condition as compared to the end of the normal walking condition (S3 Fig 6).

Stride time

For stride time, we found no significant effect of Condition, and hence, no after-effects (S3 Fig 7).

Since we did not find an after-effect for foot placement control, and no consistent after-effect for the other outcome measures, we did not proceed to test for washing-out of any after-effect.
