# Supplementary material for: Mediolateral foot placement control can be trained: Older adults learn to walk more stable, when ankle moments are constrained
Source: PLoS One. 2023 Nov 1;18(11):e0292449. doi: 10.1371/journal.pone.0292449 (PMC10619794; doi:10.1371/journal.pone.0292449)
Supplement: S3 File — (PDF) [file pone.0292449.s004.pdf]

S4 Questionnaires

As S4 we attach the translated (from Dutch to English) questionnaire participants took in the final training session.

	**1) Do you experience any positive consequences from the training? If yes, what kind of consequences?**
**S1**	Yes. I am more aware of my walking pattern. You pay attention to your steps. I corrected my old stepping habits and now point my feet more straight ahead. I am happy with it.
**S2**	Yes, my feeling says my stability improved. My muscles are also stronger.
**S3**	Not in daily life.
**S4**	Yes I am more aware of my own steps.
**S5**	Yes, I think so. I know that stability is not my strongest point. I always brush my teeth on one leg. I know it is not my strongest point, I am aware of it and I found it helpful.
**S6**	Not that I am aware of.
**S7**	No
**S8**	How do I stand on my feet?
**S9**	Yes, more focused on and more knowledge on how to place the foot. Improvement in stability. Improvement foot placement control as well as initiating the step to be placed forward. Knowing in advance where you want to place your foot. Not displacing it in the air. As much as possible initiating foot placement from the control of the lower back.<br><br>No longer afraid, [Related to spinal cord injury], to place my foot in the wrong place. Important: - activating foot muscles in such a way that balance is optimal – how to place your foot
**S10**	Not noticeable. (unfortunately)
	**2) Do you experience any negative consequences from the training? If yes, what kind of consequences?**
**S1**	No. It was all very nice for me. I didn't feel pain. It did cost some healthy exertion.
**S2**	Muscle tension on top of the foot, leg, hip [not able to translate : 's.i.g.'] Disappears after a while.
**S3**	But also no negative consequences.
**S4**	None.
**S5**	Sometimes it takes a bit of effort, you need to stay focused on how you walk. When walking with [the LesSchuh] you sweat a bit more, probably because of the need to focus.
**S6**	No.
**S7**	No.
**S8**	None.
**S9**	The day after I feel the nervous system has to recover (for example in relation to jogging).
**S10**	No (fortunately)
	**3) Did you participate in a balance training program before?**
**S1**	Not in the past year. I did participate in other experiments

**S2**	5 x week yoga which also covers balance
**S3**	No.
**S4**	No.
**S5**	No.
**S6**	I practiced some ballet dance steps for myself.
**S7**	No.
**S8**	No.
**S9**	Not in a class, but I am using 15 different balance exercises (but these are mainly for foot muscles) [The LesSchuh training] reaches out more to above.
**S10**	No.
**4) Did you or are you doing balance training at home?**	
**S1**	No.
**S2**	5 x week yoga which also covers balance
**S3**	Not specifically balance training but a lot of resistance training with weights (like standing on toes with 70 kg, which obviously requires balance)
**S4**	No.
**S5**	Brushing teeth on one leg (each morning and evening)<br>Also in athletics practice they incorporate balance training.
**S6**	See above.
**S7**	Yes, in the gym.
**S8**	No actually.
**S9**	- [see previous question]
**S10**	Yes.
**5) How much time do you spend weekly on sports/physical activity? Can you describe what kind of activities?**	
**S1**	Every day. Cycling, walking, I don't drive my car often. I want to start swimming again. I can go 45 km on an electrical bike without getting tired.
**S2**	Yoga, walking, walking the dog, cycling, making puzzles, 2 hours a day.
**S3**	3x per week 2 hours strength training.<br>Besides that cycling , skating , playing tennis and every morning 30 minutes a workout.
**S4**	3-4 times a week.
**S5**	Running and walking. 5-6 hours a week. (He is a marathon runner)
**S6**	I learned some exercises in heart rehabilitation.
**S7**	3.5 hours running, 2 hours gym.
**S8**	3 times 1 hour gym and cycling ± 100 km/week
**S9**	Intense sports each day 1.5 hour. (jogging, resistance training))<br>Walking 1.5 hours a day. On average 40 min. cycling per day, gently on a city bike. Not short.
**S10**	14 hours<br>Two times per week resistance training. An hour walking/e-bike/gardening.
**6) Did you fall or slip in the past year? If yes, how often? And how serious were the consequences?**	
**S1**	No.
**S2**	No.
**S3**	No.
**S4**	No.
**S5**	I fell max one time, but I cannot remember it.

**S6**	2x when getting of my bike, the saddle is (really) too high<br>1 x standing on a bench along the canal, which fell through the ice.
**S7**	No
**S8**	Two times, one time while walking, one time with the bike. Consequence was a sore feeling and a bruise.
**S9**	I didn't fall last year. Before that light hamstring injury during sprinting. Mid June (last year) I fell on my knee, until April knee-cap not the same. I can keep doing sports (both times I tripped across a tree trunk).
**S10**	No.
**7) Do you have any other comments related to this study?**	
**S1**	-
**S2**	Challenge. Extraordinary fun and helpful guidance. Helpful. Experiencing your own body even better.
**S3**	Easier to walk with 1 cm than with 1.5 cm ridge. 2 cm is almost the same as normal walking. After that I felt it a little.
**S4**	Side-effect is that the walking makes me think more clearly. Furthermore, it was a great pleasure to participate sweet researchers! Good luck with your research and in life!
**S5**	No, positive, fun, safety well taken care of, friendly, kind.
**S6**	No.
**S7**	No, but the last time on normal shoes felt really good.
**S8**	Professional and enjoyable guidance!
**S9**	I would like a strength/weakness analysis and advice on what to pay attention to. I know it, but I am open to it. I feel lucky that I am still trainable.
**S10**	Nice researchers ☺.
