# Supplementary material for: Mediolateral foot placement control can be trained: Older adults learn to walk more stable, when ankle moments are constrained
Source: PLoS One. 2023 Nov 1;18(11):e0292449. doi: 10.1371/journal.pone.0292449 (PMC10619794; doi:10.1371/journal.pone.0292449)
Supplement: S4 File — (PDF) [file pone.0292449.s005.pdf]

S5 Inclusion/Exclusion questionnaire

Dutch version

Checklist in-exclusie criteria oudere proefpersonen

voor deelname experimentele studies naar staan of lopen onder matige intensiteit

Instructies: Deze checklist dient, na goedkeuring van de ECB,afgenomen te worden bij alle oudere deelnemers (>65 jaar) die deelnemen aan een experimentele studie aan de FBW.

- ***expliciete exclusie criteria:***
  - worden door onderzoekers vetgedrukt aangegeven
  - kunnen leiden tot directe exclusie indien antwoord JA is
  - leeftijd en mentale gezondheid zijn altijd expliciete criteria
- ***aandachtspunten***:
  - criteria waarbij de hoofdonderzoeker obv vervolgvragen besluit tot exclusie als de veiligheid van deelnemer in het geding kan komen
- ***overige criteria***:
  - worden aangegeven met nvt

**Titel onderzoek**	Foot placement control can be trained: Older adults learn to step accurately when ankle moments are constrained
**Naam deelnemer**	
**Geboortedatum**	
**Naam onderzoeker**	
**Datum**	

Leeftijd =	Nee	Ja
**Bent u jonger dan 65 jaar?**		

Mentale gezondheid: MMSE score =	Nee	Ja
Mini Mental State Examination (MMSE) < 25 (max 30)		
**Mini Mental State Examination (MMSE) < 19**		

MMSE test (NLversie) beschikbaar via ECB website;
>24: mentaal gezond; 19-24: enige beperking; **<19: ernstig cognitief beperkt, wilsonbekwaam: exclusie!**

Antropometrie	Nee	Ja

Is uw Body Mass Index > 30 kg/m^2			Lengte:<br>Gewicht:

Hart- en vaatproblemen	Nee	Ja	Indien ja: hoe vaak en wanneer?
Heeft u ooit een hartstilstand en/of een bypass-operatie gehad?			
Heeft u wel eens pijn op de borst?			
**Heeft u ooit een herseninfarct of hersenbloeding gehad?**			
Heeft u ooit een longembolie gehad?			
Heeft u een hoge bloeddruk (bovendruk > 140, onderdruk > 90) en/of gebruikt u medicijnen tegen hoge bloeddruk?			
Heeft u een hoog cholesterolgehalte (>5.2) en/of gebruikt u medicijnen tegen een hoog cholesterolgehalte?			
Bent u de afgelopen 6 maanden wel eens flauwgevallen?			
Heeft u andere hartproblemen (bijvoorbeeld hartkloppingen, hartruis, kortademig) waarvoor u afgelopen jaar een cardioloog heeft bezocht (indien ja, waarvoor)?			

Gewrichtsaandoeningen	Nee	Ja	Indien ja: waar en sinds wanneer?
**Heeft u last van osteoporose (botontkalking) of osteo-arthrose (gewrichtsslijtage)?**			
**Heeft u last van rheumatoide arthritis (gewrichtsontsteking)?**			
**Heeft u een kunstgewricht (heup- of knieprothese)?**			

Neurologische aandoeningen	Nee	Ja	Indien ja: (waar) en sinds wanneer?
**Heeft u de ziekte van Parkinson?**			
**Heeft u wel eens last van tintelingen of gevoelloosheid in uw handen, voeten of benen**			

**(bijvoorbeeld neuropathie door diabetes)?**			
**Heeft u neurologische klachten waarvoor u afgelopen jaar een neuroloog heeft bezocht (indien ja, waarvoor)?**			

Vestibulaire aandoeningen	Nee	Ja	Indien ja: hoe vaak?
**Bent u regelmatig duizelig?**			

Letsels onderste extremiteiten	Nee	Ja	Indien ja: wanneer en in hoeverre hersteld?
**Heeft u afgelopen jaar uw been gebroken?**			
**Heeft u afgelopen jaar uw knie- of enkelbanden gescheurd?**			

Mobiliteit en valrisico	Nee	Ja	Aanvullende opmerkingen:
**Gebruikt u een hulpmiddel bij het lopen (zo ja, wat en hoe vaak)?**			
**Is het een probleem voor u om 10 minuten achterelkaar te lopen zonder te rusten (eventueel met uw loophulpmiddel)?**			
**Is het een probleem voor u om binnen één sessie 25 minuten te lopen zonder hulpmiddel maar met pauzes tussendoor?**			
Bent u afgelopen jaar gevallen (indien ja, hoe vaak)?			
Bent u het afgelopen jaar > 2 keer gevallen?			

Visus en gehoor	Nee	Ja	Aanvullende opmerkingen:
Heeft u problemen om de krant te lezen (eventueel met bril of vergrootglas)?			
Heeft u problemen om iemands gezicht te herkennen op een afstand van 4 meter (eventueel met bril)?			
Heeft u problemen om mijn vragen goed te horen?			

Medicatie	Nee	Ja	Aanvullende opmerkingen:
Heeft u afgelopen week slaappillen gebruikt?			
Heeft u afgelopen week antidepressiva gebruikt?			
Heeft u afgelopen week bètablokkers gebruikt?			

Loop comfort	Nee	Ja	Aanvullende opmerkingen:
**Ervaart u pijn als u loopt?**			
**Ervaart u meer pijn als u een langere tijd loopt?**			

English version

Checklist in-exclusion criteria older participants

for participation in experimental studies on standing or walking with moderate intensity

Instructions: After approval of the ECB, this list needs to be checked on all older adults (> 65 years) participating in an experimental study at the Faculty of Human Movement Sciences.

- ***explicit exclusion criteria:***
  - to be indicated in bold by the researchers
  - may lead to immediate exclusion if the answer is YES
  - age and mental health always in bold
- ***points of attention***:
  - criteria on which the principal investigator, based on specifications on these aspects, decides to exclude if the safety of participants cannot be warranted
- ***other criteria***:
  - can be indicated with N/A

**Research title**	Foot placement control can be trained: Older adults learn to step accurately when ankle moments are constrained
**Participant's name**	
**Date of birth**	
**Researcher's name**	
**Date**	

Age =	No	Yes
**Are you under 65 years of age?**		

Mental health: MMSE score =	No	Yes
Mini Mental State Examination (MMSE) < 25 (max 30)		
**Mini Mental State Examination (MMSE) < 19**		

MMSE test (Dutch version) available at ECB website;
>24: mentally healthy; 19-24: mild cognitive impairments; **<19: severe cognitive impairment: exclusion**

Anthropometry	No	Yes	
Is your Body Mass Index > 30 kg/m^2			Height:<br>Weight:

Cardiovascular problems	No	Yes	If yes, how often and when?
Have you ever had a heart attack and/or a bypass surgery?			
Have you ever had chest pain?			
**Have you ever had a stroke or brain hemorrhage?**			
Have you ever had a pulmonary embolism?			
Do you have high blood pressure (systolic blood pressure> 140, diastolic pressure> 90) and/or do you use drugs for high blood pressure?			
Do you have high cholesterol (> 5.2) and/or do you use medication for high cholesterol?			
Have you ever fainted the last 6 months?			
Do you have any other heart problems (eg, cardiac palpitations, souffle, or panting) for which you have visited a cardiologist in the past year (if so, which)?			

Joint disorders	No	Yes	If yes, where and since when?
**Do you suffer from osteoporosis (porous bones) or osteo-arthritis (joint degeneration)?**			
**Do you suffer from rheumatoid arthritis (joint inflammation)?**			
**Did you ever had a joint replacement (hip or knee prosthesis)?**			

Neurological disorders	No	Yes	If yes, (where) and since when?
**Do you have Parkinson's disease?**			
**Have you ever experienced tingling or numbness in your hands, feet or legs (eg diabetic neuropathy)?**			
**Do you have any other neurological symptoms for which you have visited a**			

**neurologist in the past year (if so, which)?**			

Vestibular disorders	No	Yes	If yes, how often?
**Do you often feel dizzy?**			

Lower extremities injuries	No	Yes	If yes, where and to what extend recovered?
**Did you break your leg in the past year?**			
**Did you have your knee or ankle ligaments torn in the past year?**			

Mobility and fall risk	No	Yes	Additional remarks:
**Do you use a walking aid (if so, what and how often)?**			
**Is it a problem for you to walk for 10 consecutive minutes without resting and without a walking aid?**			
**Is it a problem for you to walk 25 minutes in a single session without a walking aid, but with breaks in between?**			
Have you fallen in the past year (if yes, how often)?			
Did you fall > 2 times in the past year?			

Vision and hearing	No	Yes	Additional remarks:
Do you have problems in reading the newspaper (if necessary, with glasses or magnifying glass)?			
Do you have problems in recognizing someone's face at a distance of 4 meters (if necessary, with glasses)?			
Do you have problems in hearing my questions?			

Medication	No	Yes	Additional remarks:
Did you use sleeping pills over the last week?			

Did you use anti-depressants over the last week?			
Did you use beta-blockers over the past week?			

Walking comfort	No	Yes	Additional remarks:
**Do you experience any pain when walking?**			
**Do you experience more pain as the walking duration increases?**			
