# Supplementary material for: Mediolateral foot placement control can be trained: Older adults learn to walk more stable, when ankle moments are constrained
Source: PLoS One. 2023 Nov 1;18(11):e0292449. doi: 10.1371/journal.pone.0292449 (PMC10619794; doi:10.1371/journal.pone.0292449)
Supplement: Attachment — Submitted filename: Response to Reviewers.pdf [file pone.0292449.s006.pdf]

Thank you for reconsidering our manuscript. In this Response to Reviewers letter, I would like to respond to the comments in the previous decision letter for manuscript PONE-D-23-08003. In the rejection letter, you stated that you "do not feel that your submission meets our ethical requirements for human subjects research submissions".

You specified that **"Specifically, you have not stated in the Methods section of your manuscript text as requested when participants were recruited to the study. It is therefore not clear that you obtained ethics committee approval before beginning this study."**

Since we did obtain ethics committee approval before beginning this study and have now clarified this in het manuscript as follows:

"…ethical approval for this experiment had been granted by the ethical committee of the faculty of behavioral and movement science of the Vrije Universiteit Amsterdam (VCWE-2020-186R1, January fifth 2021). We started recruiting participants in April 2021," (manuscript lines 93-96).

We have also attached the acceptance letter of the ethical committee, dating January fifth 2021. As we have shared the data of this experiment on surfdrive (https://surfdrive.surf.nl/files/index.php/s/6fwmnAaH3hfxVCu), it can also be confirmed by looking at the timestamps of the raw data, that data collection commenced after receiving the ethical approval.

During prior submission, we have also been requested to fill out the Clinical Studies Checklist. However, we want to stress that this manuscript should not be considered as a Clinical Study. First of all, we did not include patients in this study. Our recruitment and inclusion/exclusion criteria were aimed at healthy older adults (i.e. older adults without neurological diseases or injuries), with sufficient strength and endurance to follow our training protocol. We have now attached the used inclusion/exclusion questionnaire as supplementary material 5. That we recruited healthy and fit older adults is additionally underscored by their results on the Short Physical Performance Battery (Table 1, manuscript page 13), which classified them to hold "no risk of impaired physical functioning". Participants thus did not have an apparent health concern that we tried to intervene upon. Rather than a clinical study, this study is a first pilot in translating fundamental knowledge to a potential training tool. As such, we would consider it a training study. However, we would even consider it a fundamental study in addition, as it investigates not only the training potential of the training tool, but also whether older adults still hold the potential to train their foot placement control.

Whilst we do not consider our study to be a clinical study, this manuscript does constitute human subjects research, and we understand clarifications were necessary to comply with the journal's ethical requirements. We believe our study was performed according to the ethical standards of human subject research. Hence, we have resubmitted our manuscript and hope our clarifications suffice.

Kind regards,
On behalf of all authors,

Moira van Leeuwen
